# Promoting Psychological Well-Being at Work by Reducing Stress and Improving Sleep: Mixed-Methods Analysis

**DOI:** 10.2196/jmir.9058

**Published:** 2018-10-19

**Authors:** Denny Meyer, Madawa W Jayawardana, Samuel D Muir, David Yen-Teh Ho, Olivia Sackett

**Affiliations:** 1 Department of Statistics, Data Science and Epidemiology Swinburne University of Technology Melbourne Australia; 2 Centre of Mental Health Faculty of Health, Arts and Design Swinburne University of Technology Melbourne Australia; 3 Virgin Pulse Boston, MA United States

**Keywords:** exercise, productivity, healthy lifestyle

## Abstract

**Background:**

Workplace programs designed to improve the health and psychological well-being of employees are becoming increasingly popular. However, there are mixed reports regarding the effectiveness of such programs and little analysis of what helps people to engage with such programs.

**Objective:**

This evaluation of a particularly broad, team-based, digital health and well-being program uses mixed methods to identify the elements of the program that reduce work stress and promote psychological well-being, sleep quality, and productivity of employees.

**Methods:**

Participation in the Virgin Pulse Global Challenge program during May to September 2016 was studied. Self-reported stress, sleep quality, productivity, and psychological well-being data were collected both pre- and postprogram. Participant experience data were collected through a third final survey. However, the response rates for the last 2 surveys were only 48% and 10%, respectively. A random forest was used to estimate the probability of the completion of the last 2 surveys based on the preprogram assessment data and the demographic data for the entire sample (N=178,350). The inverse of these estimated probabilities were used as weights in hierarchical linear models in an attempt to address any estimation bias caused by the low response rates. These linear models described changes in psychological well-being, stress, sleep, and productivity over the duration of the program in relation to gender and age, engagement with each of the modules, each of the program features, and participant descriptions of the Virgin Pulse Global Challenge. A 0.1% significance level was used due to the large sample size for the final survey (N=18,653).

**Results:**

The final analysis suggested that the program is more beneficial for older people, with 2.9% greater psychological well-being improvements observed on average in the case of women than men (*P*<.001). With one exception, all the program modules contributed significantly to the outcome measures with the following average improvements observed: psychological well-being, 4.1%-6.0%; quality of sleep, 3.2%-6.9%; work-related stress, 1.7%-6.8%; and productivity, 1.9%-4.2%. However, only 4 of the program features were found to have significant associations with the outcome measures with the following average improvements observed: psychological well-being, 3.7%-5.6%; quality of sleep, 3.4%-6.5%; work-related stress, 4.1%-6.4%; and productivity, 1.6%-3.2%. Finally, descriptions of the Virgin Pulse Global Challenge produced 5 text topics that were related to the outcome measures. Healthy lifestyle descriptions showed a positive association with outcomes, whereas physical activity and step count tracking descriptions showed a negative association with outcomes.

**Conclusions:**

The complementary use of qualitative and quantitative survey data in a mixed-methods analysis provided rich information that will inform the development of this and other programs designed to improve employee health. However, the low response rates and the lack of a control group are limitations, despite the attempts to address these problems in the analysis.

## Introduction

### Background

Workplace health initiatives promoting behavioral change have been recognized as vitally important strategies for improving the health of employees [1-3]. A workplace health and exercise program can be broadly defined as an intervention implemented in a workplace environment, targeting employees to improve health behaviors such as increasing physical activity, eating better, and taking care of their mental health [4]. However, a meta-analytic review has found that the effectiveness of such programs differs, with universal programs being more effective than targeted programs, one-on-one delivery being more effective than group-based classroom delivery, and with Web-based delivery and train-the-trainer delivery producing nonsignificant improvements [5]. Comparisons between Web- and paper-based workplace programs have shown similar retention rates and improvements; however, a randomized control trial (RCT) has shown higher popularity ratings for Web-based programs than for paper-based programs [6].

In an earlier meta-analytic review of workplace health promotion programs [7], 18 studies describing 21 RCT interventions found little overall effect for workplace health promotion programs (*d*=0.24, 95% CI 0.14-0.34). The effectiveness was larger in younger populations, in interventions with weekly contacts, and in studies where the control group received no health promotion. This systematic review highlights the importance of sound methodologies for statistical analyses and the handling of missing data in studies of this nature [7]. It was found that when an intention-to-treat analysis was performed, a 2.6-fold lower effectiveness was observed with a 1.7-fold lower effectiveness for studies controlling for confounders. In addition, studies of poor methodological quality reported a 2.9-fold higher effect size for workplace health promotion programs. Workplace health and exercise as well as workplace health promotion studies often tend to be observational in nature, without the luxury of a randomly assigned control group. This means that there is no way to determine if the results are a consequence of taking the program or due to the confounding factors. Moreover, attrition rates tend to be very high, making the likelihood of biased estimation results very high. In particular, more engaged participants are likely to be over-represented in the final results, providing an overoptimistic assessment of program efficacy. However, modern missing value methods have started addressing this problem [8-10]. In particular, the use of inverse probability weights (IPWs) is recommended when data are missing for large numbers of variables [8], and mixed model or hierarchical (multilevel) linear models are recommended over multiple imputation methods when repeated outcome measures are missing.

However, studies have shown that missing data in mHealth are closely linked to the level of engagement [11], suggesting that the inclusion of engagement measures in outcome studies may also help to address the issue of estimation bias caused by high levels of attrition.

### Objectives

Much of the research has focused on the overall effectiveness of workplace health and exercise programs and workplace health promotion programs rather than investigating the characteristics of more successful programs and investigating the characteristics of employees for whom such programs are more or less beneficial. It is this gap in the literature that this research attempts to fill, using a workplace health and exercise program entitled the Virgin Pulse Global Challenge (VPGC). In this study, we use modern missing value approaches to model outcomes of interest, including various measures of engagement as predictors in these models.

The evaluation of interventions usually involves a quantitative comparison of baseline and postassessment and/or follow-up performance using measures relevant to the intervention focus. However, postassessment surveys usually collect qualitative as well as quantitative data by way of open-ended questions. Although these qualitative data may be reported in a descriptive sense, there is seldom any attempt to incorporate this information into the analysis of how and why an intervention may fail or succeed. Due to the recent availability of sophisticated text mining tools, it is now possible to augment quantitative evaluations of an intervention with qualitative data using *mixed methods* [12,13]. In this paper, we use this approach with participant descriptions of the VPGC, demonstrating that mixed-methods evaluations can result in richer information for both researchers and commercial organizations alike.

In this paper, we address the following 4 hypotheses:

H1: Demographic effects will be associated with improvements in psychological well-being, sleep quality, stress, and productivity.

H2: Program module effects will be associated with improvements in psychological well-being, sleep quality, stress, and productivity.

H3: Program feature effects will be associated with improvements in psychological well-being, sleep quality, stress, and productivity.

H4: Program descriptions will be associated with improvements in psychological well-being, sleep quality, stress, and productivity.

## Methods

### Virgin Pulse Global Challenge

Virgin Pulse is a global Software-as-a-Service vendor providing several health and well-being programs designed to improve the psychological well-being of employees. The Global Challenge is one of the Virgin Pulse programs that features a team-based health and well-being challenge. The challenge consists of a 100-day virtual journey around the world, referred to as the 100-Day Journey. As part of the 100-Day Journey, employees are placed in teams of 7 individuals from their organization and provided with an activity tracker (the Pulse Device or other third-party-supported devices) and access to an app that is available through Web browsers and on mobile devices. Teams compete with one another to accumulate steps measured by their activity trackers.

The VPGC program differs from most other workplace health and exercise programs in terms of its breadth. In particular, the simultaneous focus on social, physical, and mental health is regarded as a strength of this program, which is seldom seen in other programs. In addition to promoting physical activity, the program incorporates a number of modules that focus on encouraging improvement in sleep, nutrition, and psychological well-being. The Balance module addresses mental health issues, and the Heart Age module provides 2 evaluations: a lifestyle score out of 1000 and heart age relative to real age.

The program is gamified to encourage employees to develop healthy habits through education, goal setting, and positive reinforcement using progress monitoring and achievement awards (eg, virtual trophies).

### Participants

The target population for this research was participants from all the organizations that were enrolled in the VPGC program that commenced in May 2016. Participants agreed to the use of their personal data by any agencies engaged by Virgin Pulse for the purposes of quality control. They did this when they signed up for the VPGC program on the internet [14], when encouraged to do so by their employers. The nature of this encouragement differed for each employer and is therefore not reported here. No Virgin Pulse incentives were offered to employees to participate.

The VPGC platform and its practices around data security and privacy have been externally audited and certified against the following standards: ISO 27001:2013, TRUSTe privacy seal, and General Data Protection Regulation governing data protection and privacy. The data were deidentified and password protected before being made available to the researchers and were held on university password-protected computers. Ethics approval for the evaluation of this program by the Swinburne University of Technology was obtained from the Swinburne University Research Ethics Committee (SHR Project 2017/061).

### Surveys

The data were automatically collected using 3 electronic Web-based surveys, administered on the internet as closed (password-protected) voluntary surveys. The initial Web-based survey was completed early in May 2016, the second Web-based survey was completed toward the end of the 100-Day Journey, and the final Participant Experience Survey (PES) was completed 2 weeks later. There was no randomization of items in any of these Web-based surveys and no adaptive questioning. The first Web-based survey included 28 questions and the second survey included 29 questions, all with a Likert scale (0-6) or Yes/No responses. For the PES, there were 27 questions in a variety of formats (text, multiple, and single response answers). Questions were presented to users in approximate groupings of *pages* to drive an easy and engaging completion process. All questions were voluntary, and there was no review step.

### Measures

To measure psychological well-being, this study used the independently validated World Health Organization 5-item questionnaire (WHO-5) on psychological well-being. A total of 5 simple and noninvasive questions constitute this measure of subjective psychological well-being, which has been validated as a sensitive and specific screening tool for depression. This scale was first published in 1998; it has been translated into 30 languages and used all over the world [15]. Responses to these items were used to calculate an overall score, where 0 is the “worst imaginable” and 100 the “best imaginable” psychological well-being.

In this study, the WHO-5 score was used as the primary outcome measure. Secondary outcome measures were self-assessed levels of work-related stress, sleep quality, and productivity for the last month (measured on a 0-6 ordinal scale pre- and postprogram). In all cases, higher scores indicated a more desirable state. All the above outcome measures were collected at the start (T1) and end (T2) of the VPGC program.

In the final PES (T3), an attempt was made to identify the engagement factors perceived to be particularly beneficial by the participants. In particular, participant perceptions were considered with regard to (1) the best program modules (ie, Physical Activity, Heart Age, Sleep, Nutrition, and Balance) and (2) the best program features (eg, virtual trophies, the leaderboard, individual and team challenges, and connection with colleagues). These variables were all measured on a binary scale (0 for a negative response and 1 for a positive response). In this final T3 survey, participants also provided a response to the following question: “How would you describe the Global Challenge to a friend or colleague?” As described below, these responses were used to create 25 text topic scores for each respondent, consisting of values between 0 and 1 [16].

### Response Rates

**Figure 1:**
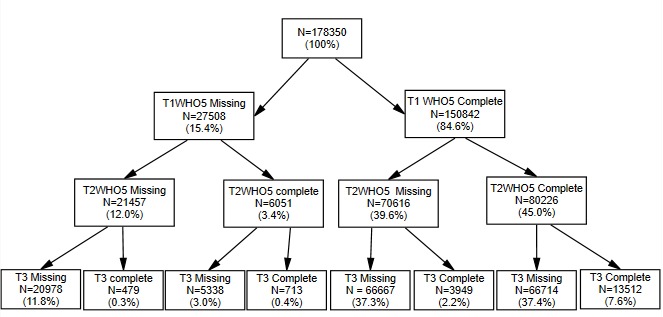
Survey participation (T1=first survey, T2=second survey, and T3=final participant experience survey). WHO-5: World Health Organization 5-item questionnaire.

Response rates often have little meaning in the context of workplace health and exercise programs and workplace health promotion programs [17] because surveys may only be partially completed. In Figure 1, the number of responses to the WHO-5 questions, representing our primary outcome measure, are summarized for the first (T1) and second (T2) surveys. A completion rate of 85% is suggested for the first survey and 48% for the second survey. The final PES (T3) was completed by only 10.5% of all participants. This low response rate suggests that there will be an estimation bias in any models fitted using the T3 data unless modern methods addressing this bias are applied. Even then, results must be viewed with caution.

### Statistical Analysis

The analysis was divided into 4 phases. In phase 1, descriptive statistics were presented for the final (T3) PES. Phase 2 involved predicting completion of this T3 survey and the WHO-5 psychological well-being measure for the second (T2) survey, using data collected in the first (T1) survey (including any missing data for the first survey). This predicted probability was inverted to produce the IPWs that were used in the ensuing analyses to reduce any estimation bias arising from missing data. Phase 3 consisted of the text mining used to produce the 25 topics and topic scores relating to the final PES question: “How would you describe the Global Challenge to a friend or colleague?” In the fourth phase, hierarchical linear model analyses were conducted for each of the outcome measures using IPW to address the problem of missing data. These analyses allow the research hypotheses to be addressed while attempting to adjust for any estimation bias caused by the low response rates. Phase 1 was conducted with IBM SPSS Version 25 software. Phase 2 and phase 3 analyses were conducted using the SAS Institute Enterprise Miner Version 14.2, whereas R software was used to produce a word cloud for the responses to the VPGC descriptions. Phase 4 was conducted using SSI Central HLM7 software.

#### Phase 1: Descriptive Statistics for Participant Experience Survey Responses

Responses for males and females were compared using independent samples *t* tests for the outcome measures and Fisher exact tests for the crosstab tests.

#### Phase 2: Estimated Inverse Probability Weights

Various tools were considered for modeling the completion of the PES (T3) and the T2 WHO-5 in terms of the first survey (T1) responses. In particular, a random forest was compared with single trees, gradient boosting, a neural network with 3 hidden nodes, support vector machines, and probit/logistic regression analyses [18-20]. All 178,350 of the original participants were randomly split into training, validation, and test datasets in a 40:30:30 ratio. To compensate for the low completion rate, the models were optimized in terms of profit, with a one unit profit for each correctly identified missing survey response and a 10 unit profit for each correctly identified completed survey response. This was done using the training data. The classification accuracy of the various tools was compared with the validation and test data to ensure reliable reproducible results. For the goodness-of-fit measure, the area under the receiver operating characteristic (ROC) curve was used for the test data, with higher values indicative of a better fit. The random forest consisted of 100 trees each constructed using a random 60% of the data, with chi-square tests used to choose the optimum splitting variable for each split. This method ensured that the random forests were not overfitted and that nonlinear relationships were accommodated. Missing values were treated as distinct categories in this analysis.

#### Phase 3: Text Mining

Text mining was applied to analyze unstructured responses to the question “How would you describe the Global Challenge to a friend or colleague?”, producing text topics [16,21]. The first step involved the extraction of terms from the text, followed by an automatic filtering of terms that were too frequent or too uncommon. A spectral decomposition was then conducted to determine which terms commonly occurred together in the same response. The most important 25 topics were extracted, and scores were then assigned to each participant for each of these topics, with values ranging from 0 to 1. Higher values indicate a more likely topic description of the VPGC.

#### Phase 4: Hierarchical Linear Model Analyses

The hypotheses were then addressed for each of the outcome measures using a hierarchical (multilevel) linear model analysis. For all these analyses, the IPWs calculated in phase 2 were applied to correct the estimation bias caused by low response rates. These hierarchical linear models [22] used all available data for the T3 sample, including cases with missing data, and they allowed for correlation between measures collected in the first 2 surveys using a maximum likelihood approach. In view of the large sample size (N=18,653), only significance levels of less than .001 are considered to be significant in the tables provided below.

## Results

### Phase 1: Descriptive Statistics for Participant Experience Survey (T3) Respondents

Table 1 shows that for the T3 respondents, the Physical Activity module was most popular, with more than 90% of participants, whereas the Heart Age module was popular with more than 60% of participants. Females appreciated the Balance module more than males, although the size of this effect was very small (*ϕ*<0.1).

**Table 1:** Gender comparison for Participant Experience Survey (T3) respondents.

Responses		Female (N=10,397)	Male (N=8256)	Total (N=18,653)	*P* value	Effect size
Age in years, mean (SD)		42.51 (10.97)	43.46 (10.44)	42.93 (10.75)	<.001	*d*=0.09
**Helpful modules, n (%)**						
	Physical Activity	9393 (90.33)	7474 (90.54)	16,867 (90.41)	.67	*ϕ*=0.00
	Heart Age	6380 (61.36)	4905 (59.41)	11,285 (60.50)	.007	*ϕ*=0.02
	Sleep	2314 (22.24)	1911 (23.15)	4225 (22.64)	.15	*ϕ*=.01
	Nutrition	3153 (30.33)	2481 (30.04)	5634 (30.20)	.68	*ϕ*=0.00
	Balance	3941 (37.90)	2765 (33.47)	6706 (35.94)	<.001	*ϕ*=0.05
**Helpful features, n (%)**						
	Mini-challenge	6731 (64.73)	4771 (57.79)	11,502 (61.66)	<.001	*ϕ*=0.07
	Leaderboard	5110 (49.12)	4429 (53.67)	9539 (51.13)	<.001	*ϕ*=0.05
	Competitions	2747 (26.41)	2471 (29.96)	5218 (27.98)	<.001	*ϕ*=0.04
	My_Location	4515 (43.43)	2855 (34.56)	7370 (39.51)	<.001	*ϕ*=0.09
	Individual mini-leagues	1875 (18.02)	1652 (20.02)	3527 (18.90)	<.001	*ϕ*=0.03
	Team mini-leagues	2316 (22.25)	1882 (22.82)	4198 (22.50)	.41	*ϕ*=0.01
	Trophies	4429 (42.59)	3194 (38.70)	7623 (40.87)	<.001	*ϕ*=0.04
	Communication sharing	738 (7.10)	480 (5.81)	1218 (6.53)	<.001	*ϕ*=0.03
	My_Stats	5951 (57.20)	4989 (60.45)	10,940 (58.64)	<.001	*ϕ*=0.03
	More colleague connect	8125 (78.90)	6512 (79.44)	14,637 (79.14)	.40	*ϕ*=0.01
**Outcomes T1, mean (SD)**						
	WHO-5^a^ (0-100)	52.86 (19.02)	56.06 (18.97)	54.27 (19.06)	<.001	*d*=0.17
	Quality of sleep (0-6)	3.20 (1.21)	3.25 (1.18)	3.22 (1.19)	.004	*d*=0.04
	Reduced work stress (0-6)	2.99 (1.38)	3.08 (1.31)	3.03 (1.35)	<.001	*d*=0.07
	Productivity (0-6)	3.85 (1.01)	3.85 (1.00)	3.85 (1.01)	.78	*d*=0.00
**Outcomes T2, mean (SD)**						
	WHO-5 (0-100)	67.70 (18.48)	69.15 (18.37)	68.33 (18.45)	<.001	*d*=0.08
	Quality of sleep (0-6)	3.99 (1.14)	4.01 (1.14)	4.00 (1.14)	.18	*d*=0.02
	Reduced work stress (0-6)	3.76 (1.43)	3.80 (1.38)	3.78 (1.41)	.11	*d*=0.03
	Productivity (0-6)	4.24 (1.01)	4.21 (1.02)	4.23 (1.01)	.04	*d*=0.03

^a^WHO-5: World Health Organization 5-item questionnaire.

**Table 2:** Pearson correlations for outcome measures at T1 and T2, with T2 correlations italicized in the Lower Triangular Matrix.

Outcomes	Psychological well-being	Quality of sleep	Reduced work stress	Productivity
Psychological well-being	—	0.452^a^	0.420^a^	0.419^a^
Quality of sleep	*0.571* ^a^	—	0.246^a^	0.207^a^
Reduced work stress	*0.533* ^a^	*0.373* ^a^	—	0.168^a^
Productivity	*0.54* ^a^	*0.392* ^a^	*0.342* ^a^	—

^a^*P*<.001.

As shown in Table 1, improved connections because of the program were claimed by 79% of the participants, suggesting that the 7-person team feature of the VPGC is particularly effective. However, there was a significant but surprisingly small association between improved connections with colleagues and endorsement of the Physical Activity module (*P*<.001, *ϕ*=.07), suggesting that shared physical activity was probably not contributing very much to this improvement in colleague connections. Other features such as the mini-challenges, the leaderboard used for comparing the performance of all teams, and personal daily step count performance (My_Stats) were also important. There were several significant but very small gender differences in the case of program feature preferences, with females favoring the mini-challenges, My_Location (virtual travel related to step count performance), and Trophy features more than males, whereas males tended to favor the competitive program features more than women.

As shown in Table 2, there were significant correlations between the outcome measures at T1 and T2, with correlations of moderate size with psychological well-being and weaker correlations between quality of sleep, reduced work-related stress, and productivity.

As shown in Table 1, on average, males scored higher than females in terms of psychological well-being (WHO-5). Males scored significantly better than females in terms of sleep quality and work-related stress only at the time of the first survey (T1). However, these effect sizes were again very small (*d*<0.2). The above descriptive statistics suggest improvements in the 4 outcome measures over the duration of the program, with large to moderate effect sizes (*η*^2^) of 0.37, 0.30, 0.23, and 0.11 for psychological well-being, sleep, work-related stress, and productivity, respectively. However, the low response rates for the T2 and particularly the T3 sample make any such claims premature. To address this issue of nonresponse bias, IPWs were calculated as indicated below, and a hierarchical (multilevel) regression analysis was conducted in phase 4.

### Phase 2: Estimated Inverse Probability Weights

Perhaps not surprisingly, the random forest produced the best results for predicting completed responses, with an area under the ROC curve of 0.719 for the test data. However, single trees were not far behind, with areas under the ROC curve of 0.704. Other methods (support vector machines, gradient boosting, neural networks, and binary regression) produced disappointing results, with areas of less than 0.62 in all cases.

**Figure 2:**
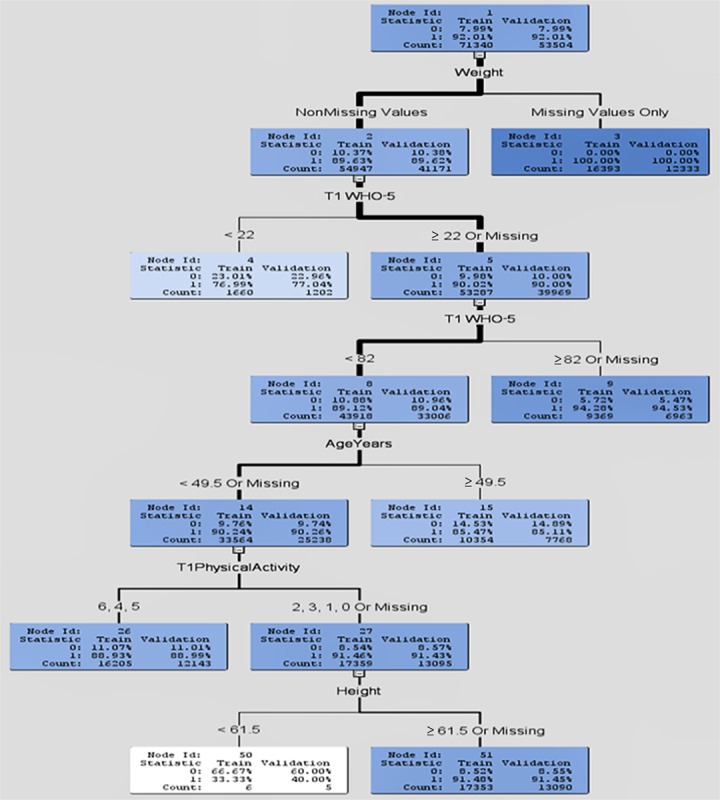
Single tree for predicting the probability for survey completion. WHO-5: World Health Organization 5-item questionnaire.

Figure 2 illustrates how probability predictions are obtained for a single (Gini) tree with splits occurring in such a way as to minimize the heterogeneity in any node in relation to survey completion. This heterogeneity is measured using the Gini criterion [20]. The “Count” in this figure refers to the number of participants for the training and validation data in each node, with the thick black line indicating the path for the majority of participants after each split. A code of 1 indicates a missing survey response, whereas a 0 indicates a completed survey response.

In the random forest and the single tree shown in Figure 2, missing values for the *weight* (kg) variables were particularly useful for identifying missing survey responses. It seems that people who refused to supply their weight in the first survey (T1) are unlikely to complete 1 or more of the last 2 surveys. Younger age (<50 years) or missing age was also highly predictive of missing survey responses. The third most important variable for the random tree was psychological well-being (WHO-5 T1). A missing or very low WHO-5 T1 score (<22) was also associated with failure to complete surveys, suggesting that people who are depressed are more likely to drop out. This result confirms that the estimation bias is inevitable if analyses are conducted on the completer data, without making any attempt to account for this bias. The inverse of the estimated completion probabilities obtained from the random forest model was therefore used as weights (IPWs) to control for bias in the hierarchical (multilevel) regression models described below.

### Phase 3: Text Mining

**Figure 3:**
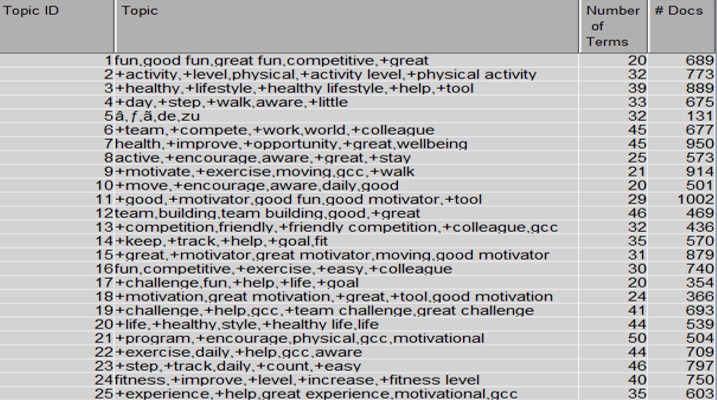
Topics extracted from the Global Challenge description.

The PES included the following question: “In one or two sentences, how would you describe the Global Challenge to a friend or colleague?” Multimedia Appendix 1 shows a word cloud for the responses to this question, whereas parsing, filtering, and topic selection produced the 25 topics displayed in Figure 3. The number of terms and the number of respondents (#Docs) associated with each topic are also displayed in Figure 3, with the highest number of respondents (1002) owning topic 11, relating to “good motivation” and “good fun.”

### Phase 4: Hierarchical Linear Model Analyses

Table 3 provides the results for independent hierarchical linear model analyses addressing each of the hypotheses. Only significant program features and text topics are included in Table 3. In terms of demographic effects, the program appears to be more beneficial for older people, and in the case of psychological well-being, it seems that women benefit more than men, with average improvements being 2.9% higher for women than men.

The program modules have significant relationships with improvements in outcomes for psychological well-being (4.1%-6.0% on average), quality of sleep (3.2%-6.9% on average), work-related stress (1.7%-6.8% on average), and productivity (1.9%-4.2% on average). However, engagement with the Physical Activity module is not significantly related to improvements in work-related stress. The Nutrition program has the strongest association with improvements in psychological well-being, the Sleep module has the strongest association with improvements in the quality of sleep, and the Balance module has the strongest association with improvements in work-related stress and productivity.

However, as shown in Table 3, only 4 of the program features are related to improvements in the outcome measures: for psychological well-being (3.7%-5.6% on average), quality of sleep (3.4%-6.5% on average), work-related stress (4.1%-6.4% on average), and productivity (1.6%-3.2% on average). Improvements in connection with colleagues, the mini-challenges, and (virtual) trophies are all associated with significant improvements in all 4 outcome measures, whereas sharing (Web-based talk) with the VPGC community is associated with significant improvements in the quality of sleep and work-related stress. There are also only 5 text topics that appear to be significantly associated with changes in the outcome measures: for psychological well-being (−3.9% to 5.2% on average), quality of sleep (−4.4% to 5.0% on average), work-related stress (−5.9% to 7.0% on average), and productivity (−1.4% to 2.7% on average).

Text topic 2 relates to physical levels of activity, and the negative association with quality of sleep suggests that participants with this perception of the program saw a decline in their quality of sleep over the duration of the program. Text topic 3 relates to a healthy lifestyle, and topic 15 relates to the perception of the VPGC as a good or great motivator. The results suggest that for people with these perceptions of the program, there was evidence of an improvement in psychological well-being and quality of sleep. Topic 20 again relates to a healthy lifestyle, suggesting that stress at work is also reduced for these people. Finally, topic 23 relates to the daily tracking of step counts, and this perception of the program is associated with negative changes in psychological well-being and stress at work.

Table 4 compares the various hierarchical linear models in terms of variance explained. The time effect refers to differences between the first 2 surveys. Clearly, this time effect explains a significant amount of the variance with *R*^2^ values ranging from 3.3% in the case of productivity to 11.9% in the case of psychological well-being. However, adding demographic variables, engagement with program modules, and features and perceptions of the program, as described to a friend or colleague, makes little difference to these *R*^2^ values, with an increase of only 2.5% for psychological well-being, 0.7% for quality of sleep, 1.4% for stress at work, and 2.2% for productivity. This means that although some support has been found for all 4 hypotheses, the size of these effects is small.

**Table 3:** Estimated program effects.

Improvements in outcome measures		Psychological well-being	Quality of sleep	Work-related stress	Productivity
T1 outcomes, range		0-100	0-6	0-6	0-6
T1, mean (SD)		54.27 (19.06)	3.22 (1.19)	3.03 (1.35)	3.85 (1.01)
**Demographic effects, estimated coefficients (% change with respect to T1)**					
	Age in years	0.069 (0.1)^a^	0.007 (0.2)^a^	0.004 (0.1)^a^	0.001 (0.0)
	Female	1.595 (2.9)^a^	0.022 (0.7)	0.055 (1.8)	0.031 (0.8)
**Module effects, estimated coefficients (% change with respect to T1)**					
	Physical activity	3.17 (5.8)^a^	0.140 (4.3)^a^	0.052 (1.7)	0.137 (3.6)^a^
	Heart age	2.24 (4.1)^a^	0.103 (3.2)^a^	0.099 (3.3)^a^	0.075 (1.9)^a^
	Sleep	3.10 (5.7)^a^	0.263 (8.2)^a^	0.157 (5.2 )^a^	0.131 (3.4)^a^
	Nutrition	3.26 (6.0)^a^	0.154 (4.8)^a^	0.132 (4.4)^a^	0.131 (3.4)^a^
	Balance	3.21 (5.9)^a^	0.222 (6.9)^a^	0.205 (6.8)^a^	0.160 (4.2)^a^
**Feature effects, estimated coefficients (% change with respect to T1)**					
	Connections	2.965 (5.5)^a^	0.172 (5.3)^a^	0.124 (4.1)^a^	0.114 (3.0)^a^
	Mini-challenge	3.012 (5.6)^a^	0.108 (3.4)^a^	0.131 (4.3)^a^	0.125 (3.2)^a^
	Trophies	2.479 (4.6)^a^	0.133 (4.1)^a^	0.146 (4.8)^a^	0.071 (1.8)^a^
	Community sharing	2.02 (3.7)	0.209 (6.5)^a^	0.195 (6.4)^a^	0.062 (1.6)
**Text topics, estimated coefficients (% change with respect to T1)**					
	#2	−0.158 (0.3)	−0.142 (4.4)^a^	−0.026 (0.9)	0.031 (0.8)
	#3	1.938 (3.6)^a^	0.127 (3.9)^a^	0.063 (2.1)	0.079 (2.1)
	#15	2.224 (4.1)^a^	0.110 (3.4)^a^	0.024 (0.8)	0.076 (2.0)
	#20	2.806 (5.2)^a^	0.160 (5.0)^a^	0.213 (7.0)^a^	0.103 (2.7)
	#23	−2.116 (3.9)^a^	−0.062 (1.9)	−0.180 (5.9)^a^	−0.054 (1.4)

^a^*P*<.001.

**Table 4:** Proportion of variance explained.

Predictors	*R*^2^ (%)			
	Psychological well-being	Quality of sleep	Work-related stress	Productivity
Time effect (T1-T2)	11.9	9.7	7.1	3.3
Time effect with demographics	12.8	9.8	7.4	3.9
Time effect with modules	12.6	10.2	7.6	4.1
Time effect with features	13.5	10.4	7.9	4.4
Time effect with text topics	12.6	10.0	7.4	4.3
All variables	14.6	10.4	8.5	5.5

^a^Psychological well-being, quality of sleep, reduced work stress, productivity.

## Discussion

### Overview

This study has identified program modules and features of a workplace health and exercise program that are particularly helpful to employees, with differences observed between men and women, and more benefit for older people. Although these gender effects are small, they are significant. Two methods have been used in an attempt to address the low response rates for the second and third surveys. A random forest has been used to create IPWs, and these weights have been utilized in hierarchical (multilevel) models, utilizing maximum likelihood methods to minimize the estimation bias resulting from missing data. The study has used text mining to incorporate qualitative data in the hierarchical linear models, which is rarely seen [23].

### Principal Findings

The research hypotheses are all supported to some extent as explained below.

1. The VPGC program is associated with greater improvements in psychological well-being, quality of sleep, and work-related stress in the case of older employees. Greater improvements in psychological well-being were found for female employees than for male employees.
2. All the modules contributed positively to psychological well-being, quality of sleep, work-related stress, and productivity, with 1 exception. The association was not significant for the Physical Activity module in the case of work-related stress.
3. Employee perceptions for 3 of the program features were significantly associated with improvements for all 4 outcome measures. These 3 features were connections with colleagues nurtured using team structures, the mini-challenges, and (virtual) trophies. VPGC community sharing (Web-based talk) was associated with improvements in quality of sleep and improved levels of work-related stress.
4. Descriptions of the program by participants provide additional context. In particular, it was found that physical activity levels had a negative association with quality of sleep, whereas daily tracking of step counts had a negative association with psychological well-being and stress at work. However, perceptions of the program as a great motivator for a healthy lifestyle were associated with improvements in psychological well-being and stress at work.

The combination of methods used in this analysis provides a better understanding of how the VPGC program may achieve behavioral change. The results suggest that although the Physical Activity module of the program is the most popular, it does not make a significant contribution to reduced work-related stress, and perhaps through its emphasis on step tracking, it has a negative association with sleep and psychological well-being as well. However, there were many positive associations, which suggest that the other modules and several of the program features are associated with positive outcomes. Perceptions of the VPGC as a tool for motivating a healthy lifestyle are especially conducive to positive outcomes.

It has been recommended that modules addressing nutrition and mental health are particularly advantageous and that a variety of program features are beneficial to address the differing preferences of men and women. The VPGC program appears to be more effective with older participants, and future work is required to explain this. However, no rigorous evaluation of the effectiveness of the VPGC is possible on account of the data limitations presented below.

### Limitations

Survey completion rates were particularly low for the final (T3) survey, which is crucial for this analysis because it contains the engagement data with the program modules and features and the description of the VPGC data used to create the text topics. With so many variables missing for 90% of the T3 data, IPW was the only way to address the threat of estimation bias. Furthermore, hierarchical linear models were needed to address the problem of missing T2 outcome values for many of the T3 respondents, ensuring that all T3 participants could be retained for the analysis. However, it is still not certain that estimation bias has been avoided. The predictors in our model (eg, helpfulness of modules and features) relate to engagement only indirectly. Other studies involving Web-based programs have used more direct engagement measures, such as the number of sessions completed [24], time to last engagement with the website, or the number of hits or time spent online [25], whereas physical activity programs have used step counts completed [26]. Future studies experiencing low response rates for final surveys should attempt to incorporate these direct measures of engagement as control variables.

Moreover, as there is no control group in this study, it is not possible to claim that the program and its individual modules are beneficial because we have no participants outside of the VPGC. In addition, none of the effects considered in this analysis were strong and must therefore be treated with caution in view of the limitations described above.

Finally, there were no follow-up data that could be used to assess the long-term effects of the program. Future studies should allow for a control group, ideally utilizing an RCT and should provide follow-up data to address these limitations.

### Comparison With Prior Work

The VPGC program is an internet-based program that previous research suggests is not the best way to conduct a workplace health and exercise program [5]. However, the results of this study indicate that for those who did complete the PES (T3), the VPGC program is a success. We have been unable to pinpoint exactly why this is the case, but it is thought that the breadth of the program is a contributing factor.

Age was identified as a significant predictor of engagement with the VPGC. Specifically, the results of this study showed that in terms of stress, sleep, and psychological well-being, the VPGC is more successful with older people. This is consistent with some previous workplace health and exercise program evaluations, which have reported that older employees tend to remain more engaged in workplace health and exercise programs in comparison with younger employees [27-30]. However, it is less consistent with other reports [7].

Results of this study confirmed that improving connections with colleagues is a particularly important feature of the VPGC. Previous mixed-methods and qualitative studies have reported that team-based workplace health and exercise programs increase motivation for exercise due to not wanting to let the team down and creating positive topics of conversation among employees [31,32]. This suggests that future workplace health and exercise programs should consider incorporating more social features for participants to engage in, in an attempt to foster greater social support among participants. Previous research has found consistent, positive associations between social support and physical activity [33]. There was a significant association between improvements in social connections and engagement with the Physical Activity module in this study, but this effect was small (*ϕ*=0.07).

Although the Physical Activity module was the most popular module, engagement with this module had no significant association with work-related stress. In addition, descriptions of the VPGC relating to physical activity had a negative association with quality of sleep, and the results suggested that the step-tracking component of the Physical Activity module might, for some participants, detract from psychological well-being. However, further investigation is required to determine why this is the case.

The Balance module, which aimed to promote psychological well-being, was found to be an important module with regard to reduced stress and enhanced sleep quality, productivity, and psychological well-being, with the Nutrition module also strongly associated with psychological well-being. This suggests that future workplace health and exercise programs may benefit from incorporating modules focusing on mental health and nutrition, rather than just targeting physical activity.

### Conclusions

According to our results for the participants who completed the final T3 survey, the VPGC program is associated with reduced work-related stress, improved quality of sleep, and improved productivity. It is also associated with increases in psychological well-being, especially in the case of women. The qualitative analysis identified a healthy lifestyle as a beneficial perception of the program, whereas the quantitative analysis indicated that the Nutrition and Balance modules contribute the most to program outcomes. However, despite the Physical Activity module being the most popular module, its contribution to reduced work-related stress appears to be limited. The social and gamified features of the program, especially the mini-challenges, appear to make the program a lot of fun.

However, these results must all be regarded as preliminary because of the lack of a control group, the low response rate for the final PES, and the lack of follow-up measures. Further work is required to provide greater certainty.

## Appendix

Multimedia Appendix 1 Word cloud for descriptions of the Global Challenge.

## Abbreviations

IPWs: inverse probability weights
PES: Participant Experience Survey
RCT: randomized controlled trial
ROC: receiver operating characteristic
VPGC: Virgin Pulse Global Challenge
WHO: World Health Organization
