# Supplementary figures and images for: Promoting Psychological Well-Being at Work by Reducing Stress and Improving Sleep: Mixed-Methods Analysis
Source: J Med Internet Res. 2018 Oct 19;20(10):e267. doi: 10.2196/jmir.9058 (PMC6231840; doi:10.2196/jmir.9058)

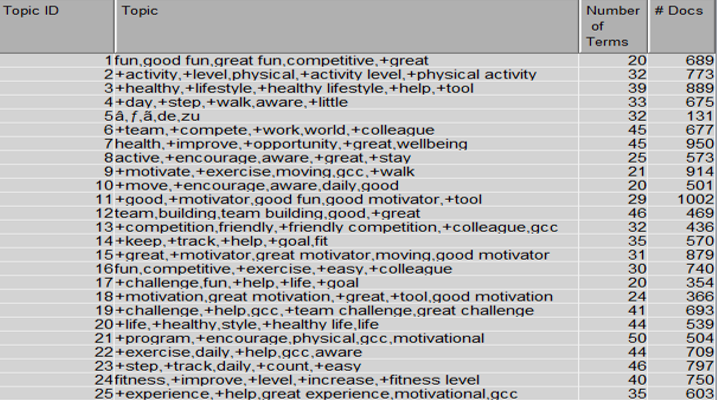

Supplement: Multimedia Appendix 1 [file jmir_v20i10e267_fig3.png]
